# Coulombic-hinderance regulation on pyrovanadates for practicable calcium-ion batteries: a solid-solution strategy

**DOI:** 10.1093/nsr/nwaf074

**Published:** 2025-02-27

**Authors:** Jun-Ming Cao, Yue Liu, Kai Li, Igor V Zatovsky, Jia-Lin Yang, Han-Hao Liu, Zhen-Yi Gu, Xuan Gao, Kai-Yang Zhang, Shuo-Hang Zheng, Xing-Long Wu

**Affiliations:** State Key Laboratory of Integrated Optoelectronics, MOE Key Laboratory for UV Light-Emitting Materials and Technology, Northeast Normal University, Changchun 130024, China; Department of Applied Physics, The Hong Kong Polytechnic University, Hong Kong 999077, China; State Key Laboratory of Integrated Optoelectronics, MOE Key Laboratory for UV Light-Emitting Materials and Technology, Northeast Normal University, Changchun 130024, China; State Key Laboratory of Rare Earth Resource Utilization, Changchun Institute of Applied Chemistry, Chinese Academy of Sciences, Changchun 130022, China; F.D. Ovcharenko Institute of Biocolloidal Chemistry, Kyiv 03142, Ukraine; State Key Laboratory of Integrated Optoelectronics, MOE Key Laboratory for UV Light-Emitting Materials and Technology, Northeast Normal University, Changchun 130024, China; Department of Chemistry, Northeast Normal University, Changchun 130024, China; State Key Laboratory of Integrated Optoelectronics, MOE Key Laboratory for UV Light-Emitting Materials and Technology, Northeast Normal University, Changchun 130024, China; Christopher Ingold Laboratory, Department of Chemistry, University College London, London WC1 H0AJ, UK; State Key Laboratory of Integrated Optoelectronics, MOE Key Laboratory for UV Light-Emitting Materials and Technology, Northeast Normal University, Changchun 130024, China; State Key Laboratory of Integrated Optoelectronics, MOE Key Laboratory for UV Light-Emitting Materials and Technology, Northeast Normal University, Changchun 130024, China; State Key Laboratory of Integrated Optoelectronics, MOE Key Laboratory for UV Light-Emitting Materials and Technology, Northeast Normal University, Changchun 130024, China; Department of Chemistry, Northeast Normal University, Changchun 130024, China

**Keywords:** calcium-ion batteries, solid-solution reinforced effect, interstitial water, layered materials, energy storage

## Abstract

Pyrovanadates are considered a promising host material for the reversible intercalation of highly charged Ca^2+^ ions due to their favorable layered structure and the presence of rich interstitial confined species. However, in calcium-ion battery (CIB) systems, the diffusion kinetics of the Ca²⁺ ions are slower, and the electrostatic interactions are stronger (compared to Li^+^), which limits the effectiveness of pyrovanadate's structural advantages. In this study, we employ an allelic reconfiguration strategy to develop novel solid-solution phase pyrovanadate materials, specifically Zn_3-x_Cu _х_ (OH)_2_V_2_O_7_·2H_2_O (x = 0, 1, 1.5). By incorporating ‘twin’ isotopic Cu elements from the adjacent *ds*-block, we activate redox reactions at non-vanadium metal sites through the modulation of electronic properties. As a result, a pronounced plateau zone during the discharge/charge process is observed. Using theoretical simulations and X-ray absorption spectroscopy, we have clarified the mechanism by which the solid solution enhances the interlayered confinement of species such as lattice water and hydroxide radicals, improving structural stability and facilitating the diffusion of highly charged Ca^2+^ ions. This approach effectively addresses the issue of layer shrinkage, which typically arises from the intense Coulombic interaction between the carrier and the host. When assembled with an active carbon anode, coin-cell CIB devices can operate steadily at a charge rate of 100 mA g^-1^ for over 1000 reversible cycles. This demonstrates the potential of innovative solid-solution design strategies to create Coulombic-force-resistant host materials for future multivalent metal-ion battery technologies, including CIB systems.

## Teaser

Novel next-generation Ca-ion batteries’ development remains stagnant, due to the high Coulombic hinderance during Ca^2+^ intercalation. As proof-of-concept, this work reveals the feasibility of solid-solution activation design on host structure, for enhancing Ca-ion storage kinetics.

## INTRODUCTION

With the rise in global electricity consumption, the continuous advancement of electrochemical energy-storage technologies is crucial for the sustainable development of human societies. As a benchmark, lithium-ion batteries (LIBs) are expected to dominate the market for portable and wearable electronic devices in the coming decade [1]. However, the challenges of limited lithium resources—due to its low crustal abundance and uneven global distribution—raise concerns about long-term supply–demand stability, cost fluctuations and safety risks. Given these issues, research efforts are increasingly focused on exploring the feasibility of multivalent metal-ion battery technologies in the post-LIB era [2,3]. Calcium ions (Ca^2+^), an earth-abundant element, hold significant potential as charge carriers in high-energy-density systems with wide operating voltage ranges. Their polarization intensity and standard reduction potential (2.87 V vs. Ca^2+^/Ca) are comparable to lithium, making them attractive alternatives to other multivalent counterparts [4–6]. However, while each Ca^2+^ ion transfers two electrons upon intercalation, its higher charge leads to stronger electrostatic interactions between the carriers and the host structure. This effect hinders the reversibility of the de-intercalation process, limiting overall battery performance. Therefore, for an ideal Ca^2+^-ion host material, structural design must incorporate sufficient open space, along with special confined species—such as functional terminations or lattice water—to mitigate the sluggish diffusion kinetics of Ca^2+^ ions [7–10].

In general, for highly charged carriers like Ca^2+^ ions, lattice water molecules can serve as structural ‘pillars’ to minimize crystal distortion during reversible intercalation reactions [9,11]. Additionally, confined H_2_O molecules contribute to electrostatic shielding, reducing Coulombic interactions between Ca^2+^ and the host lattice, as well as between pre-occupied and incoming Ca^2+^ ions. Among various materials, layered vanadates are considered promising hosts for Ca^2+^-ion storage due to their high electrochemical reactivity and favorable layered structures. Specifically, pyrovanadates such as Zn_3_Cu_х_(OH)_2_V_2_O_7_·2H_2_O, which contain both lattice water and hydroxyl (−OH) groups as interlayered confined species, exhibit several advantages. (i) A sufficiently large interlayer spacing (∼7 Å) and open diffusion pathways facilitate the accommodation of larger Ca^2+^ ions (compared to Li^+^). (ii) The synergy between interlayered H_2_O and −OH species enhances structural stability during Ca^2+^-ion diffusion while improving the reversibility of redox reactions. (iii) The high-valence state (+5) of vanadium in V_2_O_7_^4-^ groups promotes reduction reactions, delivering strong electrochemical activity similar to the widely studied V_2_O_5_ [12]. Despite these advantages, the precise functional mechanisms of lattice water and −OH groups in relation to highly charged, large-sized Ca^2+^ ions remain unclear due to the lack of systematic studies on confined species. Moreover, the limited electrochemical activity of Zn further constrains the performance benefits of layered pyrovanadates [13]. One potential approach to overcoming these limitations involves high-entropy substitution strategies, which can enhance electrode material performance to some extent. However, the co-occupation of more than five different elements introduces significant lattice distortion and diffusion hysteresis, making it difficult to ensure effective charge-carrier transport [14]. To address these challenges, we propose a solid-solution reconfiguration strategy to activate redox reactions at Zn sites in pyrovanadates, leveraging dual redox centers to enhance Ca^2+^-ion storage.

In this study, we employed an allelic reconfiguration approach by substituting Cu atoms at Zn sites, given their similar atomic size and properties. This led to the successful synthesis of a novel solid-solution phase pyrovanadate, Zn_3-x_Cu_х_(OH)_2_V_2_O_7_·2H_2_O (x = 0, 1, 1.5). The interlayer spacing remained ∼7 Å due to the negligible structural differences between Cu and Zn, providing sufficient room for the intercalation and diffusion of 2 Å Ca^2+^ ions. Using X-ray absorption spectroscopy (XAS) and density functional theory (DFT) calculations, we investigated the changes in crystal electronic properties and elucidated the fundamental mechanisms by which confined species enhance structural stability and promote Ca^2+^-ion diffusion. Based on these insights, we conducted a systematic electrochemical evaluation of equal and unequal Cu/Zn-substituted solid-solution samples to identify the optimal configuration. Furthermore, a series of *ex-situ* characterizations during electrochemical Ca^2+^-ion storage provided deeper insights into the solid-solution-induced enhancements in carrier diffusion and electrochemical performance. This study presents new opportunities for host structure design and rational material optimization in multivalent metal-ion batteries. Moreover, it affirms the feasibility of calcium-ion battery (CIB) systems using novel solid-solution pyrovanadates, paving the way for advancements in next-generation energy-storage technologies.

## RESULTS AND DISCUSSION

**Figure 1.**
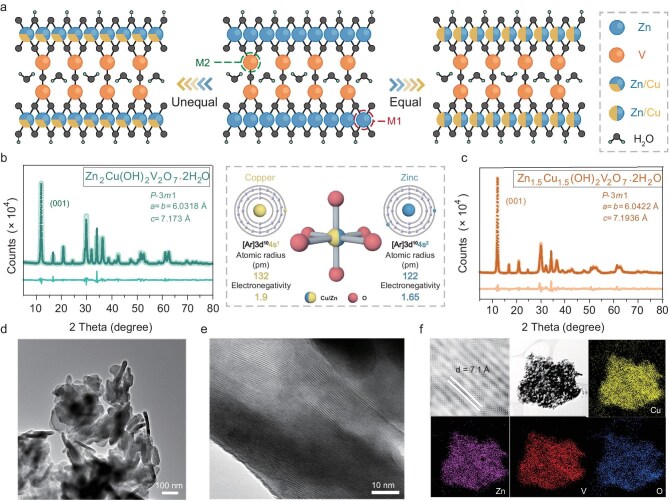
Crystal structure and morphology characterization. (a) Illustration of solid-solution substitution strategy for Zn_3_(OH)_2_V_2_O_7_·2H_2_O in unequal and equal modes. Molecular structures of pristine Zn_3_(OH)_2_V_2_O_7_·2H_2_O, solid-solution phase (Cu_1/3_Zn_2/3_)_3_(OH)_2_V_2_O_7_·2H_2_O and (Cu_1/2_Zn_1/2_)_3_(OH)_2_V_2_O_7_·2H_2_O. (b and c) The results of Rietveld refinement of XRD patterns for solid-solution phase (Cu_1/3_Zn_2/3_)_3_(OH)_2_V_2_O_7_·2H_2_O and (Cu_1/2_Zn_1/2_)_3_(OH)_2_V_2_O_7_·2H_2_O. (d) TEM image, (e) HRTEM image of (Cu_1/3_Zn_2/3_)_3_(OH)_2_V_2_O_7_·2H_2_O (USP) sample and (f) EDS mapping of prepared USP sample: Cu, Zn, V and O, respectively.

A facile reflux condensation method was employed to synthesize pyrovanadates, with further details provided in the Experimental Section in supplymentary data. Given the diverse oxidation states of V atoms at M_2_ sites, the Zn atoms at M_1_ sites—characterized by their limited redox activity—were identified as ideal targets for solid-solution reconfiguration. Consequently, substitution was carried out in both equal (1 : 1) and unequal (1 : 2) ratios (Fig. 1a). Following co-precipitation, the X-ray powder diffraction (XRD) pattern in Fig. S1 confirms that the synthesized product is well-indexed to zinc pyrovanadate (JCPDS No. 50-0570), specifically Zn_3_(OH)_2_V_2_O_7_·2H_2_O (ZnPV). Crystallographically, ZnPV adopts a *P*-3*m*1 space group within a hexagonal crystal system, imparting enhanced mechanical robustness due to its strong symmetry. However, the relatively inert redox behavior of Zn prevents full utilization of the layered structure for electrochemical performance. To address this limitation, an isotopic solid-solution substitution strategy was employed by introducing a heteroatom. To maintain the integrity of the layered structure, Cu—an element from the same *ds*-block in the periodic table—was selected for substitution in both equal and unequal ratios. This approach minimizes severe lattice distortion, which could otherwise result from significant atomic size mismatches in the solid solution. By carefully controlling the reactant ratios, we successfully synthesized unequal and equal solid-solution pyrovanadates (USP and ESP). Rietveld refinement of the XRD results, as shown in Fig. 1b and c, confirms that the obtained patterns correspond to (Cu_1/3_Zn_2/3_)_3_(OH)_2_V_2_O_7_·2H_2_O (JCPDS No. 016-0464) and (Cu_1/2_Zn_1/2_)_3_(OH)_2_V_2_O_7_·2H_2_O (JCPDS No. 016-0465), respectively. The characteristic peaks corresponding to the (001) lattice plane, aligned along the z-direction, indicate the interlayer spacing of the layered crystals. After solid-solution reconfiguration at M_1_ sites, no significant shift is observed in the (001) plane for USP and ESP, with the interlayer spacing remaining at ∼7.1 Å, which is optimal for the reversible intercalation of Ca^2+^ ions (2.0 Å). Moreover, both USP and ESP retain the hexagonal crystal structure of ZnPV, demonstrating that Cu incorporation does not disrupt the inherent layered framework. Further structural insights are provided through molecular structure illustrations in Figs S2 and S3. Unlike conventional layered materials such as transition metal oxides and MXenes, the key distinguishing features of this layered pyrovanadate include: (i) Covalently bonded adjacent layers: the crystal layers are connected via V–O–V bonds, where an oxygen atom is shared between two VO_4_ tetrahedra. (ii) Hydroxyl (−OH) terminated MO_6_ octahedra: these provide abundant active sites for hydrogen-bond (H-bond) formation. (iii) Interlayer-confined water molecules: two water molecules are present in the interlayer space, interacting with −OH groups through H-bonding [15]. The presence of both terminal functional groups (−OH) and lattice water enhances interactions within the confined interlayer space, potentially improving the material's performance as a Ca^2+^-ion host. The scanning electron microscopy (SEM) image Fig. S4 reveals that USP exhibits a nanolump-like morphology, with an average particle size of ∼200 nm and a thickness of ∼60 nm. This thin morphology enhances the exposure of electrochemically active sites, facilitating higher redox activity at the solid-solution-modified M_1_ sites. Compared to the single-phase ZnPV material (Fig. S5), the solid-solution pyrovanadates demonstrate improved textural properties conducive to efficient Ca-ion diffusion. Additionally, as shown in Fig. 1d, the transmission electron microscopy (TEM) image of USP exhibits a highly uniform flat-layered structure. This feature becomes even more apparent in the high-resolution TEM image (HRTEM) (Fig. 1e), where clear and well-defined (001) lattice fringes are observed. The interlaced lattice fringes result from the disordered stacking of USP nanolumps, and the measured lattice spacing (∼7.1 Å) aligns well with theoretical values. Elemental mapping results from energy-dispersive X-ray spectroscopy (EDS) in (Fig. 1f), confirm the homogeneous distribution of introduced Cu and intrinsic Zn in the USP sample. This uniform distribution highlights the excellent compatibility of Cu and Zn within M_1_ sites, reinforcing the structural stability of pyrovanadates while ensuring good thermal stability.

**Figure 2.**
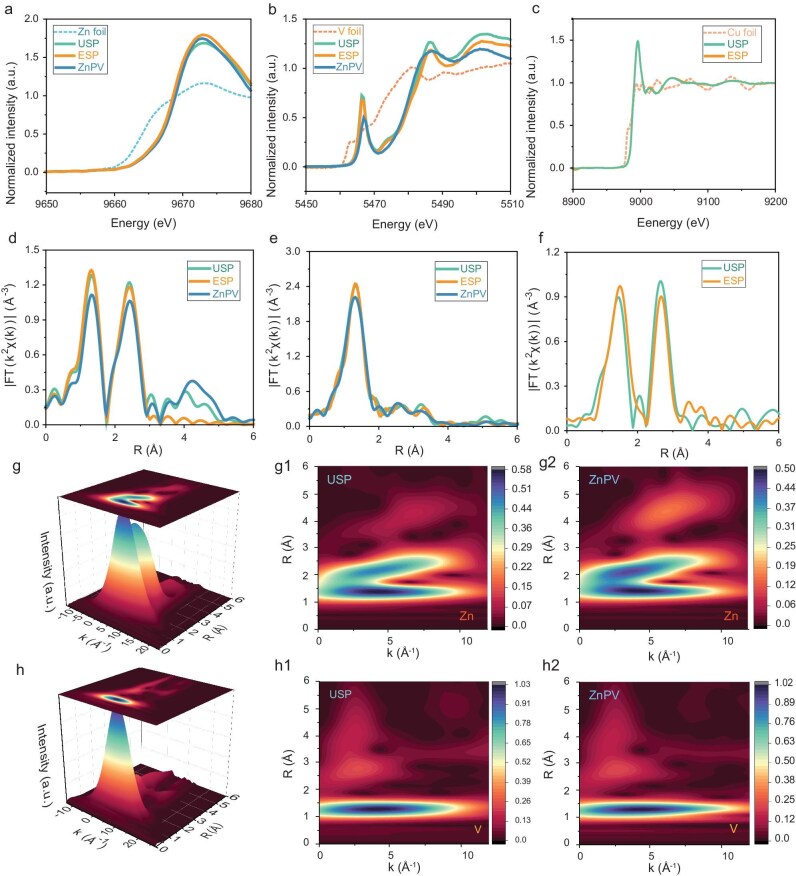
X-ray absorption spectroscopy analysis. K-edge near-edge spectrum (XANES) of (a) Zn element, (b) V element and (c) Cu element for (Cu_1/3_Zn_2/3_)_3_(OH)_2_V_2_O_7_·2H_2_O (USP), (Cu_1/2_Zn_1/2_)_3_(OH)_2_V_2_O_7_·2H_2_O (ESP) and Zn_3_(OH)_2_V_2_O_7_·2H_2_O (ZnPV) samples. Fourier transform extended fine X-ray absorption fine structure (FT-EXAFS) of (d) Zn element, (e) V element and (f) Cu element for USP, ESP and ZnPV samples. Wavelet transform K-edge EXAFS of (g) Zn and (h) V element for USP and ZnPV samples.

The introduction of Cu into M_1_ sites is expected to influence the microscopic chemical states within the ZnPV crystal. To investigate these structural changes following allelic solid-solution reconfiguration, XAS was employed. As shown in the Zn K-edge near-edge spectrum (XANES) in Fig. 2a, the threshold energy of USP and ESP is lower than that of ZnPV, indicating a decrease in Zn valence states after Cu substitution. Meanwhile, for Cu/Zn isostructural pyrovanadates, the V element exhibits a higher valence state due to charge equilibrium correction (Fig. 2b). This finding demonstrates that allelic reconfiguration at the substitution sites induces simultaneous variations in the chemical states of bonding elements. For the introduced Cu atoms, the XANES spectra in Fig. 2c show no significant differences between the unequal and equal substitution pyrovanadates, suggesting that Cu in USP and ESP exists in a similar chemical state. Further insights into coordination environments were obtained from Fourier transform (FT) analysis of extended X-ray absorption fine structure (EXAFS) spectra, which provide information on coordination numbers and bond lengths [16,17]. As shown in Fig. 2d and Table S1, the coordination number of Zn-O bonds increases after isotopic Cu-induced reconfiguration. Consequently, this leads to an uneven local charge distribution, enhancing electrostatic repulsion between Zn and O atoms, which in turn increases the Zn-O bond length. A similar trend is observed for V-O bonds, although the increase in both coordination number and bond length is less pronounced (Table S2). This can be attributed to the crystal structure: V atoms in VO_4_ tetrahedra are coordinated with four O atoms, whereas the Cu/Zn co-occupied M_1_ sites form MO_6_ octahedra coordinated with six O atoms, as discussed earlier. Therefore, during allelic reconfiguration, differences in electrostatic interactions lead to the observed variations in coordination behavior. Additionally, the increased coordination number suggests a reduced charge carrier constraint, as Cu substitution decreases V atom vacancies. This structural modification is expected to enhance Ca^2+^ ion diffusion within the interlayer spacing. As shown in Fig. 2f, the EXAFS spectrum for Cu-O bonds exhibits negligible differences in coordination number or bond length, indicating that Cu is well-incorporated into the ZnPV lattice in both USP and ESP. To further analyze local structural changes, wavelet transform (WT) EXAFS was performed on Zn and V elements, as shown in Fig. 2g and h, and Fig. S6. The maximum intensity at ∼1.5 Å in R-space shifts slightly upward, corresponding to a mild elongation of M-O bonds after allelic reconfiguration, in agreement with the FT-EXAFS analysis. Meanwhile, the WT-EXAFS spectra of V-O and Cu-O bonds show no noticeable shifts (Fig. S7), confirming that the crystal structure remains robust during isotopic Cu doping in pyrovanadates.

Conventional entropy-driven strategies for synthesizing high-entropy materials often result in severe lattice distortions and sluggish ion diffusion, due to the presence of multiple heteroatoms with significantly different sizes and electronic structures. This creates challenges for long-term cyclic stability and intercalation reversibility. However, as evidenced by the coordination fitting data in Tables S1 and S3, the coordination numbers of Zn-O and Cu-O bonds remain largely unchanged after solid-solution reconfiguration, indicating excellent compatibility between Zn and Cu. The reconfiguration process can therefore be regarded as an equivalent substitution, which enhances crystal stability while maintaining entropy-driven structural benefits. An ideal solid-solution substitution should increase configurational entropy without inducing severe lattice distortion. This principle guided the selection of Cu for allelic reconfiguration with Zn. Specifically, Cu and Zn have similar electronic structures, with fully occupied 3d orbitals. The key difference is that the 4s orbital of Cu is only partially occupied, allowing for potential Cu^+^ formation, which enhances redox activity and improves Ca^2+^ storage capabilities.

**Figure 3.**
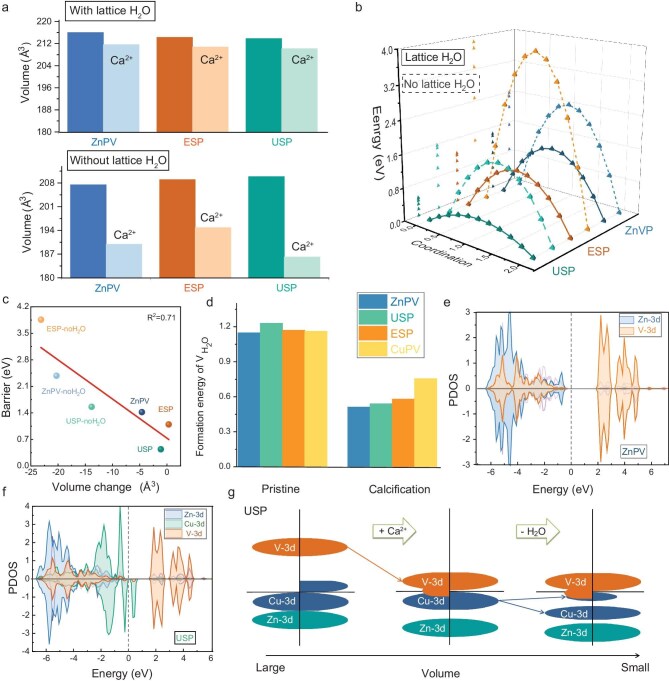
Theoretical simulation based on density functional theory (DFT). (a) Lattice volume for (Cu_1/3_Zn_2/3_)_3_(OH)_2_V_2_O_7_·2H_2_O (USP), (Cu_1/2_Zn_1/2_)_3_(OH)_2_V_2_O_7_·2H_2_O (ESP) and Zn_3_(OH)_2_V_2_O_7_·2H_2_O (ZnPV), at the condition of the existence of lattice water and Ca^2+^ ions intercalation. (b) Diffusion energy profiles of three samples with and without lattice water, (c) Linear relationship between lattice volume and diffusion energy barriers. (d) Formation energy values of H_2_O vacancies (${V_{{{\mathrm{H}}_{\mathrm{2}}}{\mathrm{O}}}}$) with and without the Ca^2+^ ions intercalation. (e and f) Partial densities of states (PDOS) patterns of pristine ZnPV and USP, including Zn-3d, V-3d and Cu-3d orbitals, respectively. (g) Mechanism illustration on electronic structure variation with Ca^2+^ ions intercalation and removal of lattice water.

Layered materials governed by van der Waals (VdW) interactions often face challenges such as disordered restacking and interlayer expansion during charge-carrier intercalation due to weak VdW forces. In the case of multivalent charge carriers like Ca^2+^, the electrostatic interactions between adjacent crystal layers are significantly stronger than those of Li^+^ or Na^+^, making Ca^2+^ diffusion more difficult. To address this issue, introducing confined interlayer species could be a viable solution. Fortunately, after allelic reconfiguration, the interlayer lattice H_2_O molecules and −OH terminations remain well preserved. DFT calculations were employed to investigate this effect. As shown in Fig. 3a, the lattice volume of solid-solution isotopic ESP and USP samples shows negligible contraction with increasing Cu content, indicating structural stability, consistent with previous analyses. When Ca^2+^ intercalates into the interlayer spacing between adjacent crystal layers, the lattice volume remains almost unchanged in Cu-containing samples. In contrast, in the absence of interlayer H₂O molecules, the lattice undergoes mild expansion due to the lack of H-bond stabilization. Furthermore, during Ca^2+^ intercalation, all ZnPV, USP and ESP samples experience abnormal crystal collapse caused by the strong electrostatic interactions of divalent Ca^2+^ ions. This suggests that H_2_O molecules act as Janus species, playing a dual role: (i) offsetting strong electrostatic interactions to maintain sufficient interlayer spacing, and (ii) restraining lattice expansion caused by heteroatomic co-occupation. This mechanism can be regarded as the essence of the ‘pillar effect’ exerted by interlayer lattice H_2_O molecules. To further explore the impact of lattice H_2_O on Ca^2+^ diffusion, Fig. 3b illustrates diffusion pathways for ZnPV, USP and ESP samples under two conditions: with and without lattice H_2_O. Evidently, samples containing lattice H_2_O exhibit significantly lower energy barriers, demonstrating their superior Ca^2+^ capabilities compared to those lacking interlayer-confined species. This suggests that lattice H_2_O also acts as a shield, mitigating strong electrostatic interactions between Ca^2+^ and the host crystal, thereby facilitating the migration of divalent charge carriers with high charge density. Moreover, based on the energy profiles, the unequal solid-solution phase (Cu_1/3_Zn_2/3_)_3_(OH)_2_V_2_O_7_·2H_2_O exhibits the lowest energy barrier, whereas the equal-substitution pyrovanadate (Cu_1/2_Zn_1/2_)_3_(OH)_2_V_2_O_7_·2H_2_O, which contains a higher Cu ratio, shows a relatively higher energy barrier. This phenomenon may be attributed to the fact that equal Cu/Zn reconfiguration at M₁ sites enhances lattice symmetry, thereby strengthening electrostatic interactions, which negatively impact Ca^2+^ diffusion. Notably, the calculated energy barriers exhibit a linear correlation with lattice parameters (Fig. 3c), highlighting the effectiveness of lattice H_2_O in alleviating lattice shrinkage in host materials. Furthermore, the coordinated interlayer H_2_O molecules significantly enhance reversible Ca^2+^ ion diffusion, further validating their role in structural stability and charge transport efficiency.

The previous discussion focused on the significance of lattice H_2_O molecules in stabilizing the structure and facilitating Ca^2+^ diffusion. However, further investigation is needed to understand how heterotopic allelic reconfiguration influences lattice H_2_O retention. Compared to Zn, Cu exhibits higher redox activity due to its variable valence states. More importantly, its similar atomic radius minimizes lattice distortion during solid-solution substitution. To ensure stable and efficient Ca^2+^ storage, it is crucial to maintain the presence of coordinated H_2_O molecules within the interlayered spacing, even when introducing heteroatoms with high electrochemical reactivity. To explore this, the formation energies of lattice H_2_O vacancies (${V_{{{\mathrm{H}}_{\mathrm{2}}}{\mathrm{O}}}}$) in ZnPV, USP and ESP samples, with and without Ca^2+^ intercalation, are summarized in Fig. 3d. The results indicate that lattice H_2_O formation levels remain relatively stable under normal conditions. However, upon Ca^2+^ intercalation, significant differences emerge. Specifically, Zn_3_(OH)_2_V_2_O_7_·2H_2_O exhibits the highest ${V_{{{\mathrm{H}}_{\mathrm{2}}}{\mathrm{O}}}}$ value, suggesting that Zn atoms effectively lock H_2_O molecules in place. As Cu content increases, ${V_{{{\mathrm{H}}_{\mathrm{2}}}{\mathrm{O}}}}$ values gradually decrease, indicating a higher tendency for coordinated H_2_O molecules to escape, which could weaken the crystal structure. Therefore, while Cu introduction is beneficial, excessive amounts may compromise structural stability. This suggests that the unequal solid-solution phase (Cu_1/3_Zn_2/3_)_3_(OH)_2_V_2_O_7_·2H_2_O is an optimal choice, balancing high redox activity and structural robustness. This phenomenon, referred to as the solid-solution reinforcement effect, establishes an equilibrium between highly reversible Ca^2+^ diffusion and effective H_2_O retention, aligning with the diffusion energy barrier analysis.

To further understand the influence of lattice H_2_O on isotopic solid-solution crystal structures, we analyzed its impact on electronic properties. Notably, the intrinsic electronic structure remains largely unchanged, regardless of H_2_O presence, except for a slight reduction in band gap, which enhances electronic conductivity (Fig. S8). Upon Cu substitution at M_1_ sites, the partial density of states (PDOS) near the Fermi level increases significantly (Fig. 3e and f), indicating a weakening of Coulombic repulsion between adjacent crystal layers. As a result, PDOS localization decreases, and electronic conductivity improves. Specifically, the band gap reduces from 2.1 eV (ZnPV) to 0.82 eV (isotopic samples). As illustrated in Fig. S9, this confirms that Cu incorporation enhances electronic conductivity. However, Cu's sensitivity to lattice H_2_O also makes lattice contraction inevitable. Therefore, a controlled Cu/Zn ratio is crucial for realizing the synergistic solid-solution reinforcement effect. For the USP and ESP samples, the V atoms in the anionic V_2_O_7_^4−^ groups also exhibit high electrochemical activity. To examine this further, we analyzed the impact of lattice H_2_O on V-3d orbitals under conditions with and without Ca^2+^ intercalation (Figs S11 and S12). The results indicate that V-3d orbitals remain unaffected by lattice H_2_O, suggesting that H_2_O mainly interacts with M_1_ sites in the solid-solution structure. However, upon Ca^2+^ intercalation, the electronic structure of Cu-3d and V-3d orbitals changes significantly. Specifically, the PDOS at the Fermi level increases, weakening electrostatic repulsion and reducing electron constraints, which in turn accelerates electron transfer during Ca^2+^ intercalation reactions. Notably, in the equal substitution phase, the enhancement of electronic conductivity is relatively lower, leading to stronger Ca^2+^ constraints compared to unequal substitution counterparts. This further supports the conclusion that only an optimal Cu content enhances reversible Ca^2+^ intercalation. Additionally, when Ca^2+^ ions insert into the ZnPV host, the PDOS for the V-3d orbital remains highly localized, indicating strong constraints on Ca^2+^ migration. This suggests that the electrostatic interactions in ZnPV limit its effectiveness as a Ca^2+^ host, further emphasizing the advantages of controlled Cu/Zn solid-solution substitution.

The variation in electronic structure under different conditions—lattice H_2_O presence and Ca^2+^ intercalation—is vividly illustrated in Fig. 3g. Taking USP as an example, when Ca^2+^ ions intercalate into the interlayered space, the Fermi level shifts upward, bringing the Cu-3d and V-3d orbitals closer together, significantly reducing the band gap. As a result, the USP host exhibits an enhanced charge transport capability, facilitating an electron-conductivity-dominated charge transfer process. In contrast, when lattice H_2_O is removed, the interlayered electrostatic repulsion increases, causing energy level splitting in the Cu-3d orbital. Consequently, the valence band beneath the Fermi level loses its continuity, indicating a localized electronic structure. This leads to strong confinement of Ca^2+^ ions within the interlayer space, severely limiting electron migration. Structurally, the absence of lattice H_2_O triggers crystal collapse, effectively trapping Ca^2+^ ions irreversibly upon intercalation, thereby preventing their reversible insertion. This phenomenon is further confirmed by charge density difference analysis (Fig. S10), which illustrates the structural degradation occurring during Ca^2+^ intercalation in the absence of H_2_O molecules. Additionally, the partially unoccupied Cu-3d orbital originates from energy level overlap between Cu and in-plane O atoms, due to the presence of −OH groups. In the equal substitution scenario, with higher Cu content, the Fermi level also shifts upward to some extent. However, a direct band gap remains between Cu-3d and V-3d orbitals, signifying that while charge carrier transport improves, excessive Cu incorporation hinders Ca^2+^ diffusion. This aligns with previous conclusions (Fig. S13). From a structural perspective, the introduction of Cu induces Jahn-Teller distortion in MO_6_ octahedrons. Notably, when the Cu/Zn ratio reaches 1 : 1, the distortion becomes more pronounced, which negatively impacts Ca^2+^ reversible insertion [18]. Therefore, an optimal Cu content is crucial for enhancing Ca^2+^ storage reactivity.

**Figure 4.**
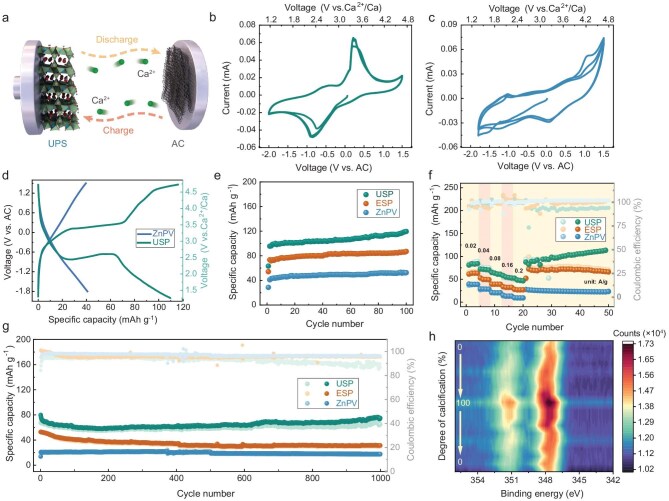
Investigation into electrochemical Ca^2+^ ions storage behaviors. (a) Schematic illustration on coin-cell configuration for electrochemical evaluation. (b and c) CV curves at a scanning rate of 0.2 mV s^−1^ for the initial three cycles for (Cu_1/3_Zn_2/3_)_3_(OH)_2_V_2_O_7_·2H_2_O (USP) and Zn_3_(OH)_2_V_2_O_7_·2H_2_O (ZnPV) samples. (d) Galvanostatic charge-discharge (GCD) branches for USP and ZnPV samples at a charge rate of 0.02 A g^−1^. (e) Cyclic stability at a current density of 0.02 A g^−1^. (f) Rate capabilities and (g) long-term cyclic stability at a current density of 0.1 A g^−1^ for USP, (Cu_1/2_Zn_1/2_)_3_(OH)_2_V_2_O_7_·2H_2_O (ESP) and ZnPV samples. (h) *Ex-situ* high-resolution Ca 2p XPS spectra at various charge and discharge states during Ca^2+^ reversible intercalation.

Building on the insights from lattice H_2_O effects and solid-solution reinforcement in USP and ESP samples, the electrochemical behavior of reversible Ca^2+^ storage was evaluated. Figure 4a presents the systematic electrochemical measurements conducted in a coin-cell two-electrode system, where Kurary activated carbon serves as both the counter and reference electrodes, with a 0.5 M Ca(TFSI)_2_ in dimethyl ether (DME) electrolyte [19–21]. The cyclic voltammetry (CV) curves for USP, ESP and ZnPV samples at a sweep rate of 0.2 mV s^−1^ are displayed in Figs 4b, S12 and 4c, respectively. Clearly, the solid-solution samples exhibit reversible Ca^2+^ intercalation redox reactions across a broad voltage range of −2.0 V to 1.5 V (vs. AC). The strong current responses at −0.9 V (cathodic) and 0.3 V (anodic) correspond to the reversible intercalation of high-charge-density Ca^2+^ ions. Compared with the CV curve of ZnPV Fig. 4c, it is evident that Cu incorporation activates Ca^2+^ intercalation electrochemical reactions. In contrast, ZnPV—where all M_1_ sites are fully occupied by Zn—lacks prominent redox peaks in the −1.8 V to 1.5 V range, indicating that Zn alone is insufficient to facilitate Ca^2+^ intercalation. As a pyrovanadate, the V atoms in V_2_O_7_ groups exhibit multi-valence properties, but their contribution to Ca^2+^ storage performance is limited due to the presence of inactive Zn. Nevertheless, ZnPV can still store Ca^2+^ ions reversibly, albeit with inferior electrochemical performance, as confirmed by higher energy barriers from DFT calculation. Notably, a pair of broad redox peaks in all samples corresponds to vanadate-based materials, confirming the involvement of V atoms in redox reactions during Ca^2+^ storage. Furthermore, CV curves at different scan rates (Figs S14 and S15) illustrate the high reversibility of charge transfer and ion diffusion processes in USP and ZnPV samples. The galvanostatic charge-discharge (GCD) curves of USP and ZnPV are shown in Fig. 4d. As expected, the USP sample exhibits distinct voltage plateaus at −0.9 V and 0.3 V, which align well with the CV results, confirming its robust redox activity. In contrast, the GCD profiles of ZnPV display a continuous slope rather than clear plateaus, indicating poor Ca^2+^ intercalation-induced redox activity. The well-defined voltage plateaus and strong redox peaks observed in the USP host provide further evidence for the solid-solution reinforcement effect. This effect arises from Cu incorporation, which enhances the synergy between Cu and V atoms, thereby improving electrochemical reactivity for Ca^2+^ intercalation into the confined interlayer space. This enhancement is attributed to reduced diffusion barriers and a narrower band gap, as confirmed by the electronic structure analysis. Additionally, GCD profiles at varying current densities (Figs S16 and S17) were examined to evaluate the reversibility of redox reactions during Ca^2+^ intercalation. Under different current densities, the discharge/charge plateaus remain well-preserved, with only minor polarization effects observed. This further validates the solid-solution effect in stabilizing the redox reaction process.

The long-term electrochemical stability of the samples was evaluated through extensive cycling tests. Figure 4e compares the specific capacity for Ca^2+^ ions (C_Ca-ions_) storage of USP, ESP and ZnPV samples under continuous operation at a charge rate of 0.02 A g^−1^. Over the first 100 cycles, all three hosts demonstrate excellent cycling stability, although their C_Ca-ions_ values differ. Due to the Cu-induced solid-solution reinforcement effect, both USP and ESP exhibit enhanced Ca^2+^ storage capabilities, with C_Ca-ions_ of 119.5 mAh g^−1^, 87.1 mAh g^−1^ and 52.5 mAh g^−1^ for USP, ESP and ZnPV, respectively. The initial lower C_Ca-ions_ values of three samples were presumably ascribed to that, the battery operation was begun with the Ca^2+^ ions intercalation (discharging states from open circuit voltage, OCV) into the pristine crystal hosts, resulting in insufficient ion diffusion. Following confirmation of the electrochemical stability at low rates, the charge storage behavior under dynamic charge rates was analyzed (Fig. 4f). The C_Ca-ions_ of USP gradually decreases from 84.7 mAh g^−1^ at the current density of 0.02 Ag^−1^ to 47.5 mAh g^−1^ at 0.2 A g^−1^. However, upon returning to the initial low charge rate, the Ca^2+^ storage capacity recovers almost to its original level, confirming the excellent rate capability of all three samples. The rapid and reversible Ca^2+^ intercalation in Zn_3-х_Cu_x_(OH)_2_V_2_O_7_·2H_2_O (х = 0, 1, 1.5) is likely driven by synergistic interactions between Cu and V elements with variable valence states, in agreement with the Cu-induced solid-solution reinforcement mechanism discussed earlier. Once the storage capacity and rate performance were verified, the long-term high-rate stability was investigated. Figure 4g presents the specific capacities of USP, ESP and ZnPV at a higher current density of 0.1 A g^−1^ over 1000 cycles in assembled CIBs. The C_Ca-ions_ values remain above 75 mAh g^−1^ for unequal substitution samples, outperforming their fully substituted counterparts. The capacity decay remains within an acceptable range, demonstrating that all three samples effectively store high-charge-density divalent Ca^2+^ ions. This long-term stability is primarily attributed to lattice H_2_O-induced interlayer confinement, which enables stable and reversible Ca^2+^ storage. To further verify the reversible intercalation of Ca^2+^ ions during electrochemical cycling, *ex-situ* X-ray photoelectron spectroscopy (XPS) was conducted to analyze the chemical states of Ca in the USP sample (Fig. 4h). Distinct Ca 2p peaks at ∼347 eV and 351 eV correspond to the Ca 2p_3/2_ and 2p_1/2_ orbitals, respectively [10]. Notably, during a full Ca^2+^ intercalation and de-intercalation cycle, the intensity of the Ca 2p peaks fluctuates reversibly as the calcification degree increases and decreases. This serves as direct evidence of Ca^2+^ ions’ active participation in the charge storage process and confirms their highly reversible intercalation into the host structure.

**Figure 5.**
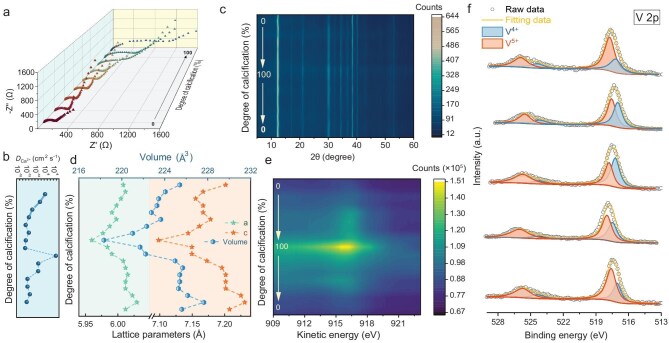
Analysis of *ex-situ* Ca^2+^ ions diffusion behaviors for USP host. (a) Electrochemical impedance spectroscopy (EIS) during Ca^2+^ ions intercalation process. (b) Ca^2+^ ions diffusion coefficients (${D_{{\mathrm{C}}{{\mathrm{a}}^{{\mathrm{2 + }}}}}}$) calculated based on the above EIS spectra. (c) XRD patterns, (d) corresponding lattice parameters of *a, c* and *V*. (e) Auger photoelectron spectroscopy (APS) for Cu 2p orbitals within a complete Ca^2+^ ions intercalation and de-intercalation process. (f) High-resolution V 2p XPS spectra during Ca^2+^ ions intercalation process.

With the Ca^2+^ ion storage mechanism confirmed, the next step was to investigate the diffusion behavior of Ca^2+^ ions within the host lattice. This was analyzed using *ex-situ* electrochemical impedance spectroscopy (EIS) to determine the Ca^2+^ diffusion coefficient (${D_{{\mathrm{C}}{{\mathrm{a}}^{{\mathrm{2 + }}}}}}$). Figure 5a presents the EIS spectra for USP at various Ca^2+^ insertion states, with Nyquist plots shown in the inset. The diffusion coefficient (${D_{{\mathrm{C}}{{\mathrm{a}}^{{\mathrm{2 + }}}}}}$) was calculated using the equations: ${D_{{\mathrm{C}}{{\mathrm{a}}^{{\mathrm{2 + }}}}}}$ = R^2^T^2^/2a^2^n^4^F^4^C^2^σ^2^ and Z′ = R_s_ + R_ct_ + σω^−1/2^. The values of ${D_{{\mathrm{C}}{{\mathrm{a}}^{{\mathrm{2 + }}}}}}$ are correlated only with Warburg factor σ, mathematically, which could be yielded from the slope between Z′ versus the minus square root of dynamic frequencies (ω^−1/2^). The corresponding fitting circuits are shown in Fig. S18 [22,23]. The ${D_{{\mathrm{C}}{{\mathrm{a}}^{{\mathrm{2 + }}}}}}$ values decrease as the degree of calcification (DOC) increases, indicating a continuous Ca^2+^ intercalation process. The primary reason for the declining diffusion ability is the gradual occupation of interlayer sites by pre-inserted Ca^2+^ ions, leading to stronger electrostatic repulsion, making subsequent Ca^2+^ intercalation increasingly difficult. As a result, ${D_{{\mathrm{C}}{{\mathrm{a}}^{{\mathrm{2 + }}}}}}$ drops to the range of 10^−14^ and 10^−15^ cm²/s. Conversely, under de-intercalation conditions ${D_{{\mathrm{C}}{{\mathrm{a}}^{{\mathrm{2 + }}}}}}$ jumps to ∼10^−6^ cm²/s, as previously intercalated Ca^2+^ ions rapidly migrate out of the structure due to weak electrostatic constraints. As more Ca^2+^ ions exit the interlayer space, ${D_{{\mathrm{C}}{{\mathrm{a}}^{{\mathrm{2 + }}}}}}$ declines again near the completion of the de-intercalation process (Fig. 5b). Additionally, the EIS spectra during DOC reduction are provided in Fig. S19, where the reversible shifts further confirm the high diffusion kinetics and excellent reaction reversibility of Ca^2+^ ions.

The electrostatic strain and phase transition behavior during reversible Ca^2+^ intercalation into the interlayer space of USP remains unclear, especially considering that Ca^2+^ ions, as divalent carriers, possess high charge density. To investigate this, *ex-situ* XRD patterns across the full DOC were analyzed (Fig. 5c). The characteristic (001) diffraction peaks remain nearly unchanged, confirming that the USP sample retains its hexagonal crystal structure throughout Ca^2+^ intercalation and de-intercalation, without undergoing any phase transition. This structural stability is crucial for highly reversible Ca^2+^ storage (the XRD pattern at the OCV state of the USP cathode is shown in Fig. S20). To further examine these structural effects, lattice parameters (a, c) and unit cell volumes were calculated based on the *ex-situ* XRD data. As shown in Fig. 5d, the a-axis parameter remains nearly constant, varying by only 0.1 Å, indicating that dislocation slip after Ca^2+^ insertion is unlikely. Given the layered structure of USP, changes along the z-axis are particularly significant for understanding lattice expansion or collapse. Unlike the a-axis, the c-axis parameter exhibits a minor contraction (∼0.13 Å, ∼1.8% strain) after Ca^2+^ intercalation [24]. This slight structural contraction in the z-direction aligns with DFT calculations, which suggests that interlayer lattice water plays a critical role in maintaining structural robustness during Ca^2+^ diffusion. As a result, the lattice volume fluctuations remain within 9.3 Å^3^, ensuring a strain-free process throughout battery cycling in USP//AC CIBs. These strain-free and phase-stable properties contribute significantly to the long-term cyclic stability of the system.

In isotopic Zn_3-х_Cu_x_(OH)_2_V_2_O_7_·2H_2_O (х = 0, 1, 1.5) samples, both Cu and V serve as active redox centers. To investigate their role, we analyzed their chemical states using *ex-situ* XPS at different charge and discharge states. For Cu, *ex-situ* Auger photoelectron spectroscopy (APS) was conducted for the USP cathode (Fig. 5e). The prominent peak at ∼914.5 eV (kinetic energy) indicates the emergence of monovalent Cu^+^, suggesting that pristine Cu^2+^ ions are chemically reduced via electron transfer from inserted Ca^2+^ ions [25]. As DOC increases (i.e. during full Ca^2+^ intercalation) the intensity of the Auger peak rises, confirming the progressive reduction of Cu^2+^ to Cu^+^. Conversely, when the battery operates in a Ca-free state, the APS peak intensity decreases, indicating the reoxidation of Cu^+^ to Cu^2+^. These findings validate the electrochemical activation of M_1_ sites due to Cu incorporation, which is a crucial aspect of the solid-solution reinforcement effect. Since vanadium atoms in vanadate anionic groups frequently serve as electrochemically active centers, we performed *ex-situ* XPS to examine changes in the high-resolution V-2p spectra (Fig. 5f and Figs S21–S23). The peak at ∼517 eV corresponds to the V 2p_2/3_ orbital and can be deconvoluted into components associated with V^5+^ and V^4+^ with different binding energies. From the perspective of DOC growth, the intensity of high-resolution XPS peaks of V^4+^ with lower binding energy become more intensive, which means the electron transfer also occurred on M_2_ sites occupied V atoms, during Ca^2+^ ions intercalation and de-intercalation process [26,27]. In summary, both Cu and V function as dual redox centers, facilitating reversible Ca^2+^ diffusion. The synergistic interaction between these elements enhances charge accommodation, effectively supporting high-energy-density Ca^2+^ storage. This interplay serves as the key underlying mechanism driving the solid-solution reinforced electrochemical performance of these materials.

## CONCLUSIONS

In this work, we employed an allelic reconfiguration strategy to synthesize isostructural Zn_3-х_Cu_x_(OH)_2_V_2_O_7_·2H_2_O (х = 0, 1, 1.5) pyrovanadates, incorporating both equal and unequal cationic substitutions. The resulting materials exhibit similar structural properties, with a robustly bonded interlayer space stabilized by V–O–V bridges. Notably, interlayer-confined species, including lattice water and –OH groups, contribute to pillar and electrostatic shielding effects, which facilitate the reversible intercalation of large-sized Ca^2+^ ions with high charge density. The introduction of electrochemically active Cu into Zn-occupied sites provides multiple advantages. (i) Electronic structure modulation—Cu substitution reduces the band gap, thereby enhancing intrinsic electronic conductivity. (ii) Structural stability—the optimized formation energy of ${V_{{{\mathrm{H}}_{\mathrm{2}}}{\mathrm{O}}}}$ in isostructural crystals ensures strong lattice water anchoring, maintaining structural integrity under high Ca^2+^ storage conditions. (iii) Enhanced redox activity—the activation of dual redox centers (Cu and V) significantly improves reversible Ca^2+^ intercalation, boosting overall electrochemical performance. This solid-solution reinforcement mechanism enables a high-capacity Ca^2+^ storage process with minimal strain and no phase transitions. Furthermore, XAS and DFT calculations provide fundamental insights into the mechanism of isostructural substitution, while systematic *ex-situ* characterization validates the structure–property correlations underlying our allelic reconfiguration strategy. Our findings offer a new perspective on the rational design of layered materials for the high-performance storage of multi-valent ions, including Ca^2+^ ions, paving the way for next-generation energy-storage solutions.

## Supplementary Material

nwaf074_Supplemental_File
