# Supplementary material for: Coulombic-hinderance regulation on pyrovanadates for practicable calcium-ion batteries: a solid-solution strategy
Source: Natl Sci Rev. 2025 Feb 27;12(5):nwaf074. doi: 10.1093/nsr/nwaf074 (PMC12023857; doi:10.1093/nsr/nwaf074)
Supplement: nwaf074_Supplemental_File [file nwaf074_supplemental_file.pdf]

Supporting Information

Coulombic-hinderance regulation on pyrovanadates for practicable calcium-ion batteries: a solid-solution strategy

Jun-Ming Cao[a,f], Yue Liu[a], Kai Li[b,*], Igor V. Zatovsky[c], Jia-Lin Yang[a], Han-Hao Liu[d], Zhen-Yi Gu[a], Xuan Gao[e], Kai-Yang Zhang[a], Shuo-Hang Zheng[a], Xing-Long Wu[a,d,*]

[a] MOE Key Laboratory for UV Light-Emitting Materials and Technology, Northeast Normal University, Changchun 130024, P. R. China
[b] State Key Laboratory of Rare Earth Resource Utilization, Changchun Institute of Applied Chemistry, Chinese Academy of Sciences, Changchun 130022, P. R. China
[c] F.D. Ovcharenko Institute of Biocolloidal Chemistry, NAS Ukraine, 42 Acad. Vernadskoho blv., 03142 Kyiv, Ukraine
[d] Department of Chemistry, Northeast Normal University, Changchun 130024, P. R. China
[e] Christopher Ingold Laboratory, Department of Chemistry, University College London, WC1H0AJ, London, United Kingdom
[f] Department of Applied Physics, The Hong Kong Polytechnic University, Hong Kong, P. R. China

* Correspondence authors, E-mails: likai@ciac.ac.cn; xinglong@nenu.edu.cn

Materials

As for single phase zinc pyrovanadate $Zn_3(OH)_2V_2O_7 \cdot 2H_2O$ preparation, 3 mM NH_4VO_3 was dissolved in 100 mL deionized water using round-bottom flask, heating at 90 during reflux condensing process in an oil bath for pre dissolution. After solution was clear, another solution containing 50 mL 0.1 M $Zn(NO_3)_2$ was added under continuous stirring, following by a reaction time for 24 hours. The as-prepared powder could be obtained after drying at 60℃. For solid-solution phase materials, the same preparation process is applied, only the amount of $Zn(NO_3)_2$ and $Cu(NO_3)_2$ is in equal (1:1) and unequal (2:1) ratios, $(Cu_{1/2}Zn_{1/2})_3(OH)_2V_2O_7 \cdot 2H_2O$ and $(Cu_{1/3}Zn_{2/3})_3(OH)_2V_2O_7 \cdot 2H_2O$, respectively. For electrode preparation, the Ti foil is chosen for current collectors, which were coated by electrode slurry consisting of mixture containing three active materials, Ketjen black conductive agent and PVDF binder, in a mass ratio of 8:1:1. The electrolyte used is 0.5 M $Ca(TFSI)_2$ in dimethoxyethane (DME). Besides, the Kurary active carbon is used as both counter and reference electrode materials.

Materials Characterizations

The detailed crystal structure and properties were performed by XRD (RIGAKU, D/MAX 2550V), SEM (Megellan 400), TEM (Tecnai G2 F20 S-TWIN 200 kV), and XPS (Kratos, AXIS SUPRA+). XAFS was carried out using the RapidXAFS 2M by transmission (or fluorescence) mode at 20 kV and 20 mA. The electrochemical performance was evaluated by electrochemical workstation (CHI660E) and cyclic test instrument (NEWARE CT-4000).

Computational Details

The calculations were carried out using density functional theory with the PBE form of generalized gradient approximation functional (GGA).[1] The Vienna ab-initio simulation package (VASP)[2-5] was employed. The plane wave energy cutoff was set as 400 eV. The Fermi scheme was employed for electron occupancy with an energy

smearing of 0.1 eV. The first Brillouin zone was sampled in the Monkhorst−Pack grid.[6] The 3×3×1 k-point mesh for the surface calculation. The energy (converged to 1.0 $\times 10^{-6}$ eV/atom) and force (converged to 0.01eV/Å) were set as the convergence criterion for geometry optimization. The spin polarization was considered in all calculation.

The transition state (TS) structures and the reaction pathways were located using the climbing image nudged elastic band (CI-NEB) method.[7] The minimum energy pathway was optimized using the force-based conjugate-gradient method until the maximum force was less than 0.03 eV/Å.

Reference

(1) Perdew, J. P.; Burke, K.; Ernzerhof M. Generalized Gradient Approximation Made Simple. *Phys. Rev. Lett.* **1996**, 77, 3865-3868.

(2) Kresse, G.; Furthmüller, J. Efficiency of Ab-initio Total Energy Calculations for Metals and Semiconductors Using a Plane-Wave Basis Set. *Comp. Mater. Sci.* **1996**, 6, 15-50.

(3) Kresse, G.; Hafner, J. Ab initio Molecular Dynamics for Liquid Metals. *Phys. Rev. B* **1993**, 47, 558-561.

(4) Kresse, G.; Hafner, J. Ab initio Molecular-Dynamics Simulation of the Liquid-Metal–Amorphous-Semiconductor Transition in Germanium. *Phys. Rev. B* 1994, 49, 14251-14269.

(5) Kresse, G.; Furthmüller, J. Efficient Iterative Schemes for Ab Initio Total-Energy Calculations Using a Plane-Wave Basis Set. *Phys. Rev. B* **1996**, 54, 11169-11186.

(6) Monkhorst, H. J.; Pack, J. D. Special Points for Brillouin-Zone Integrations. *Phys. Rev. B* **1976**, 13, 5188-5192.

(7) Henkelman, G.; Uberuaga, B. P.; Jónsson, H. A Climbing Image Nudged Elastic Band Method for Finding Saddle Points and Minimum Energy Paths. *J. Chem. Phys.* 2000,113, 9901-9904.

(8) Grimme, S. Semiempirical GGA-type Density Functional Constructed with A Long-Range Dispersion Correction. *J. Comput. Chem.* 2006, 27, 1787–99.

(9) Grimme, S.; Antony J.; Ehrlich S.; Krief H. A Consistent and Accurate Ab Initio Parametrization of Density Functional Dispersion Correction (DFT-D) for the 94 elements H–Pu. *J. Chem. Phys.* 2010, 132,154104.

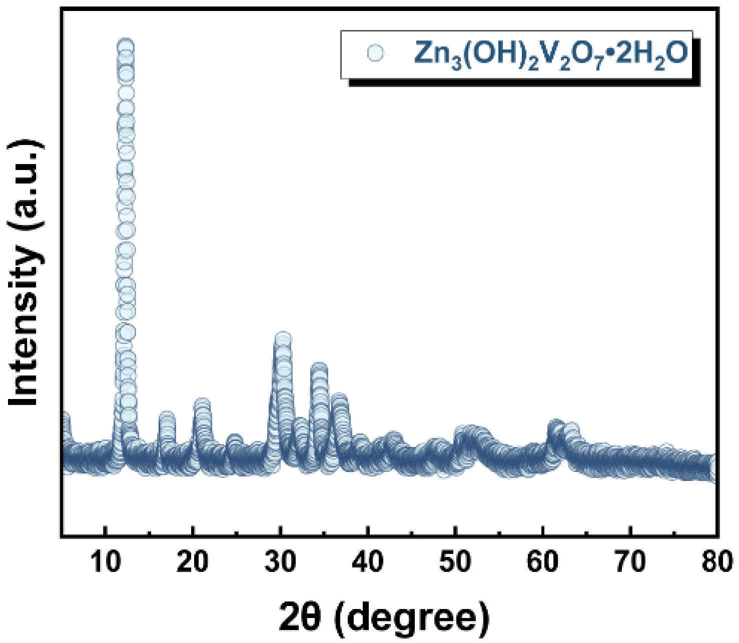

.**Figure S1.** XRD patterns of $Zn_3(OH)_2V_2O_7{\cdot}2H_2O$.

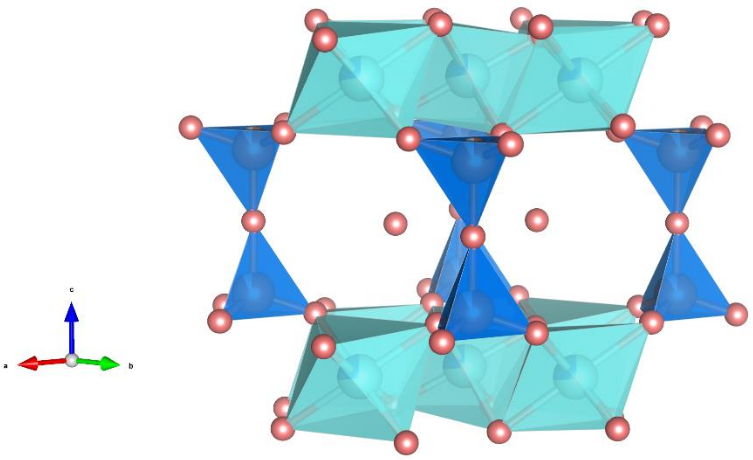

Figure S2. Molecular structure illustration: $(Cu_{1/3}Zn_{2/3})_3(OH)_2V_2O_7 \bullet 2H_2O$ sample.

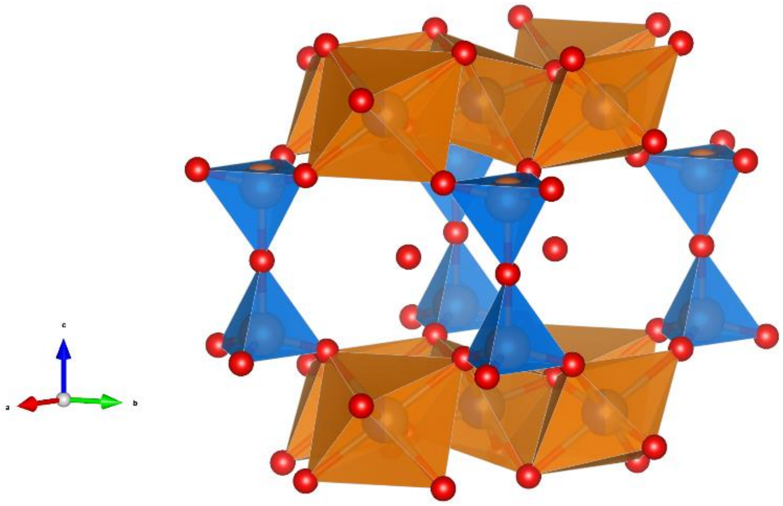

Figure S3. Molecular structure illustration: $(Cu_{1/2}Zn_{1/2})_3(OH)_2V_2O_7{\bullet}2H_2O$ sample.

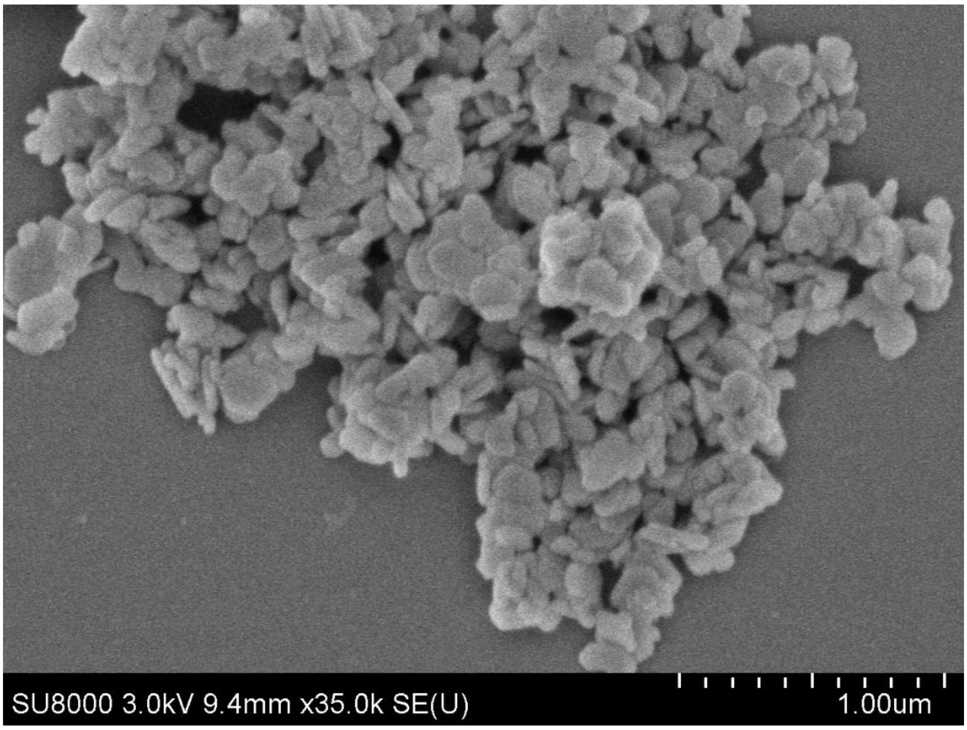

Figure S4. SEM image of solid-solution phase pyrovanadates USP sample.

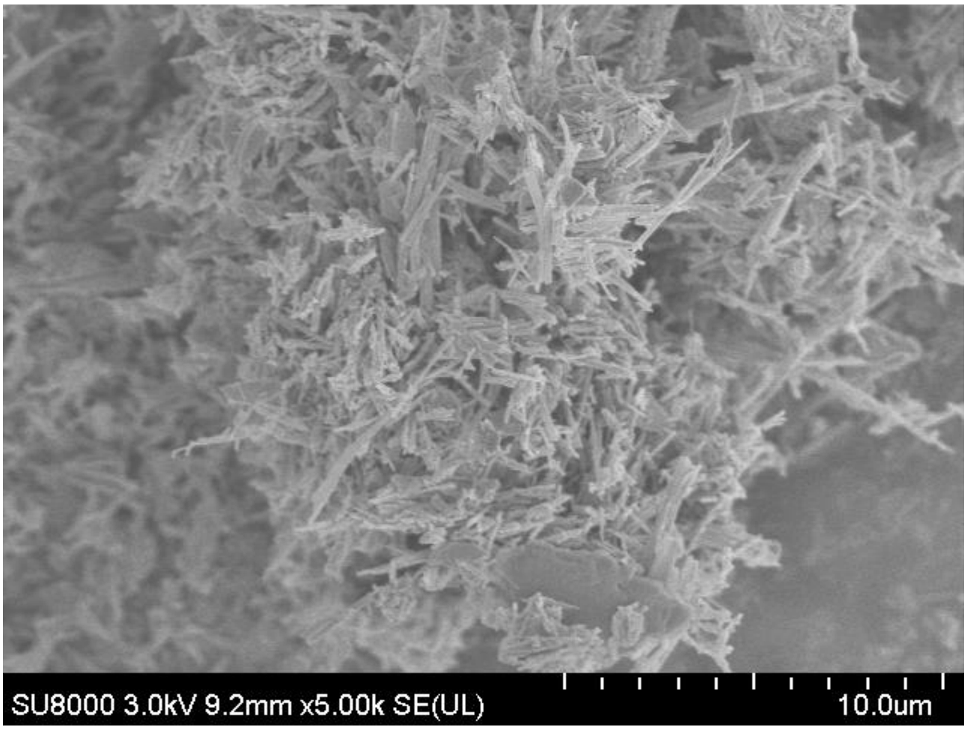

Figure S5. SEM image of single phase pyrovanadates ZnPV sample.

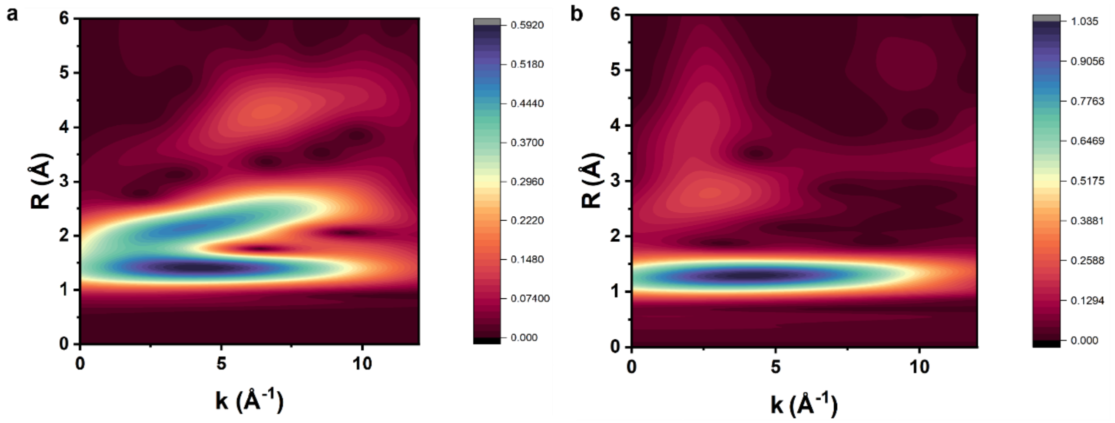

Figure S6. Wavelet transforms K-edge EXAFS. (*a*) Zn and (*b*) V element in $(Cu_{1/2}Zn_{1/2})_3(OH)_2V_2O_7{\bullet}2H_2O$ sample.

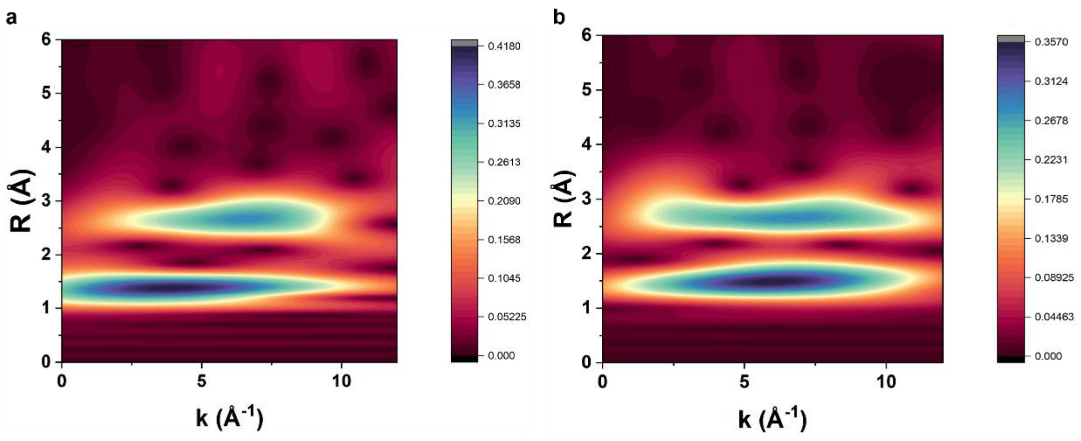

Figure S7. Wavelet transforms K-edge EXAFS. Cu element in (*a*) $(Cu_{1/3}Zn_{2/3})_3(OH)_2V_2O_7{\bullet}2H_2O$ and (*b*) $(Cu_{1/2}Zn_{1/2})_3(OH)_2V_2O_7{\bullet}2H_2O$ samples.

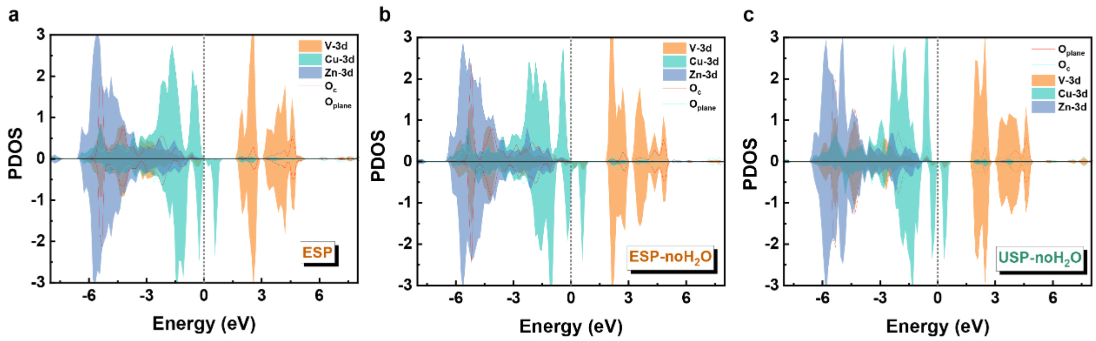

Figure S8. Partial density of states. (*a*) $(Cu_{1/2}Zn_{1/2})_3(OH)_2V_2O_7{\bullet}2H_2O$ and (*b*) $(Cu_{1/2}Zn_{1/2})_3(OH)_2V_2O_7$ and (*c*) $(Cu_{1/3}Zn_{2/3})_3(OH)_2V_2O_7$ samples.

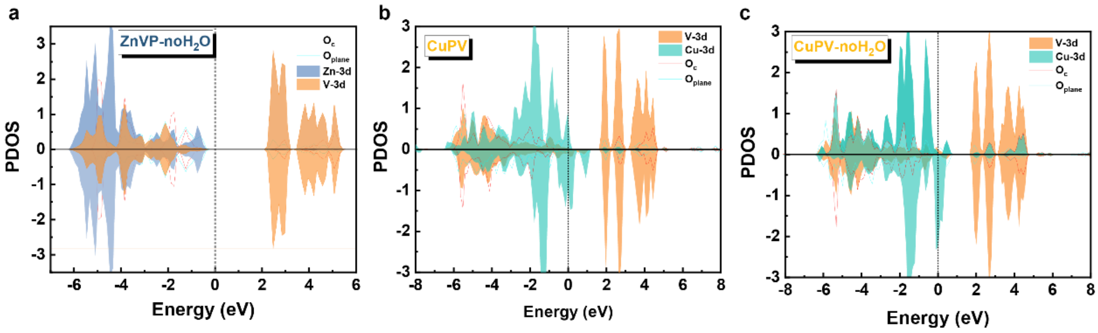

Figure S9. Partial density of states. (*a*) $Zn_3(OH)_2V_2O_7$ and (*b*) $Cu_3(OH)_2V_2O_7 \cdot 2H_2O$ and (*c*) $Cu_3(OH)_2V_2O_7$ samples.

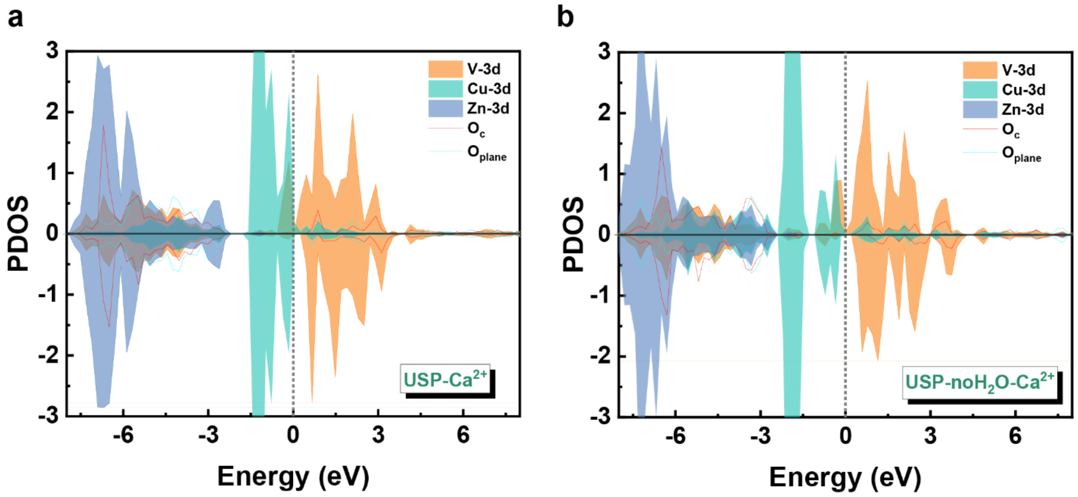

Figure S10. Partial density of states. (*a*) $(Cu_{1/3}Zn_{2/3})_3(OH)_2V_2O_7$ •$2H_2O$ with Ca^{2+} ions intercalation and (*b*) $(Cu_{1/3}Zn_{2/3})_3(OH)_2V_2O_7$ with Ca^{2+} ions intercalation.

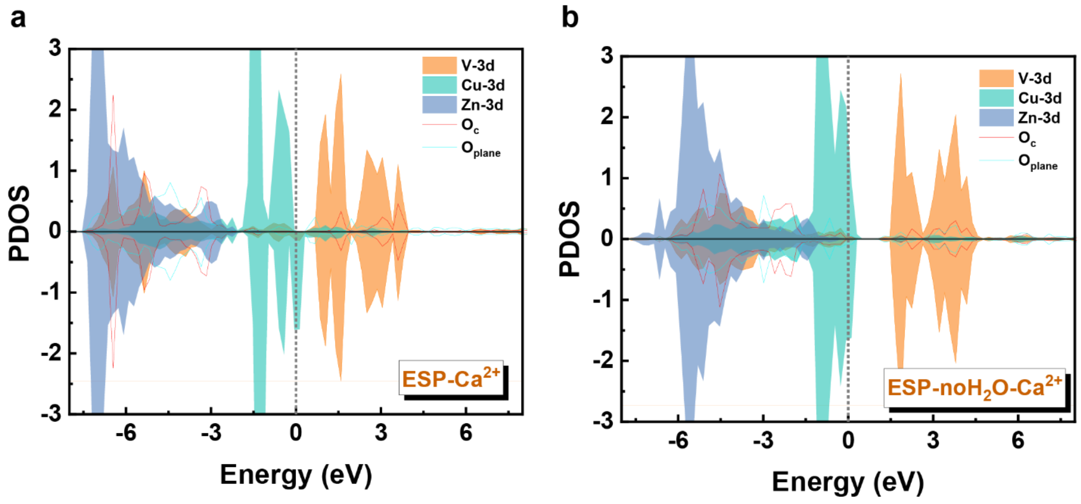

Figure S11. Partial density of states. (*a*) $(Cu_{1/2}Zn_{1/2})_3(OH)_2V_2O_7$ •$2H_2O$ with Ca^{2+} ions intercalation and (*b*) $(Cu_{1/2}Zn_{1/2})_3(OH)_2V_2O_7$ with Ca^{2+} ions intercalation.

a

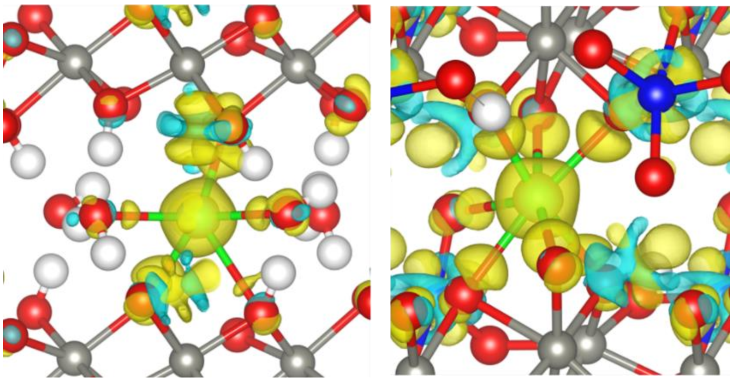

b

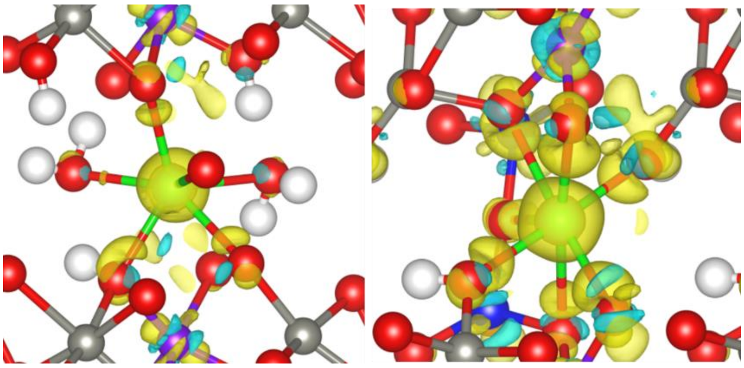

c

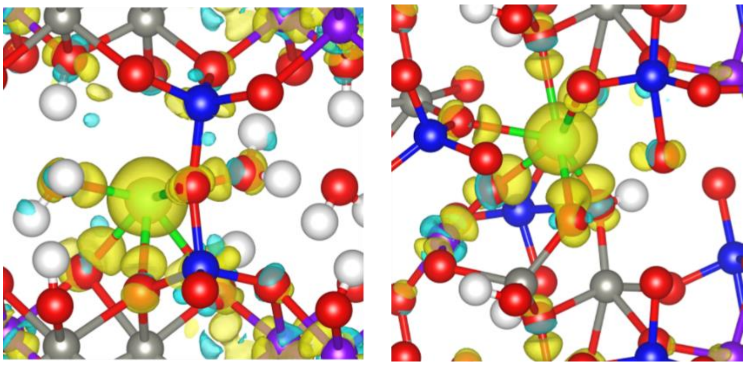

Figure S12. Charge density differences for Ca^{2+} ions intercalation at the conditions with and without lattice water. (*a*) $Zn_3(OH)_2V_2O_7$ •$2H_2O$, (*b*) $(Cu_{1/3}Zn_{2/3})_3(OH)_2V_2O_7$ •$2H_2O$ and (*c*) $(Cu_{1/2}Zn_{1/2})_3(OH)_2V_2O_7$ •$2H_2O$

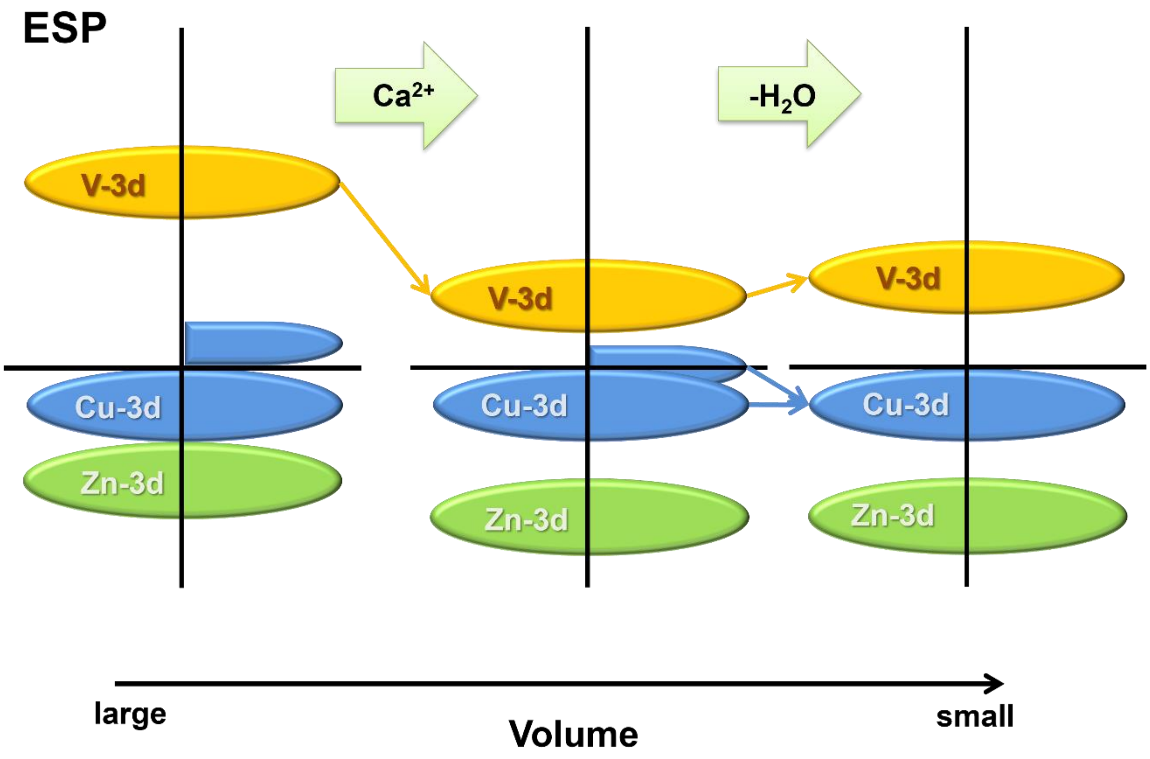

Figure S13. Mechanism illustration on electronic structure variation with Ca^{2+} ions intercalation and removal of lattice water for $(Cu_{1/2}Zn_{1/2})_3(OH)_2V_2O_7 \cdot 2H_2O$ sample.

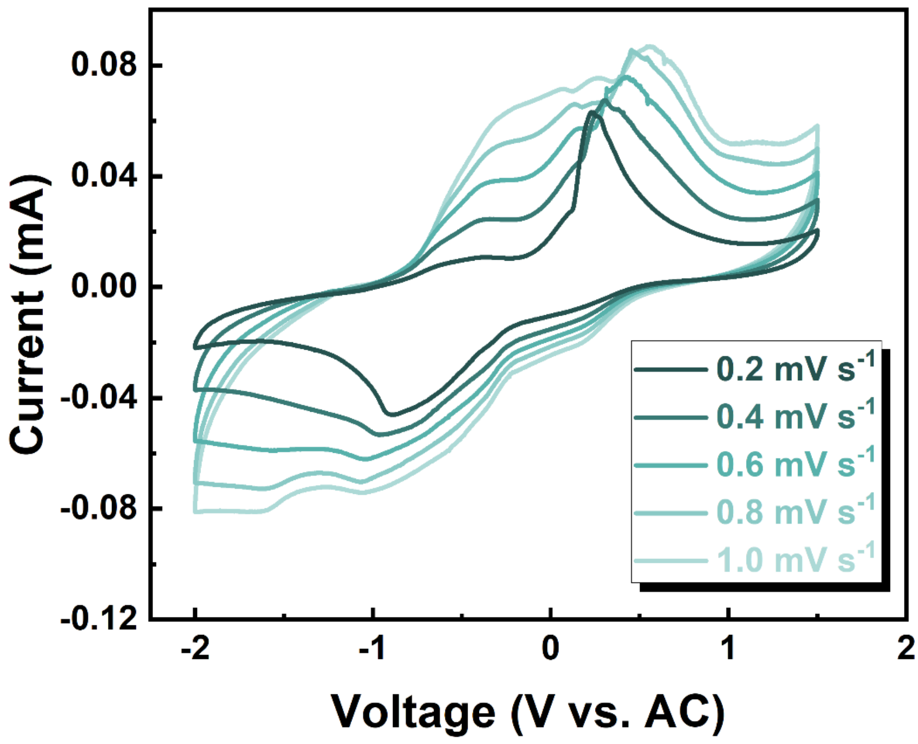

Figure S14. CV curves at different scan rates for solid-solution pyrovanadates USP sample.

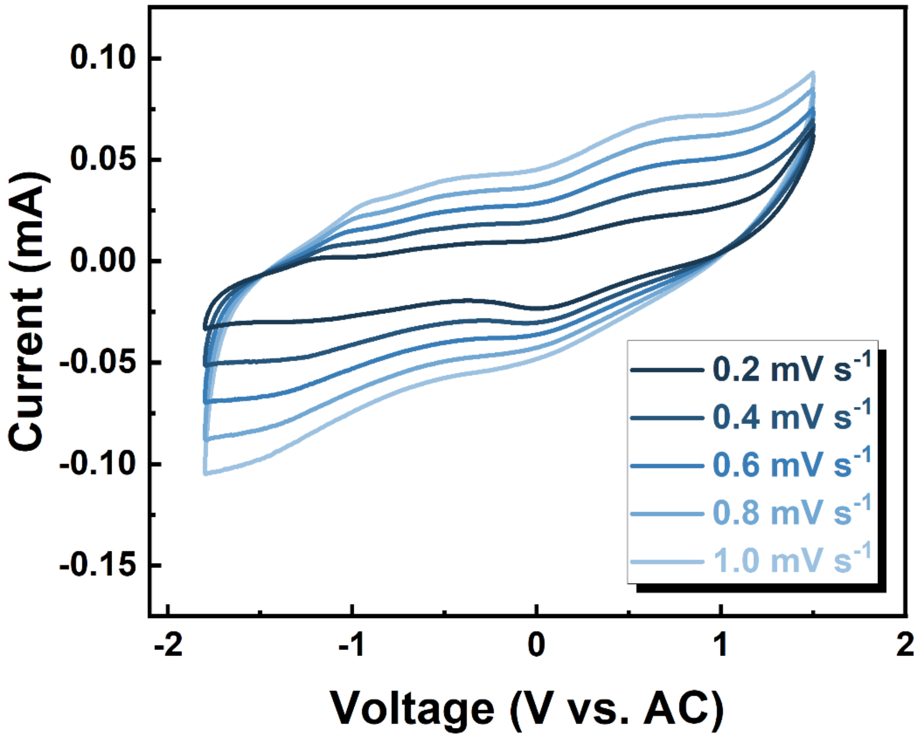

Figure S15. CV curves at different scan rates for single phase pyrovanadates ZnPV sample.

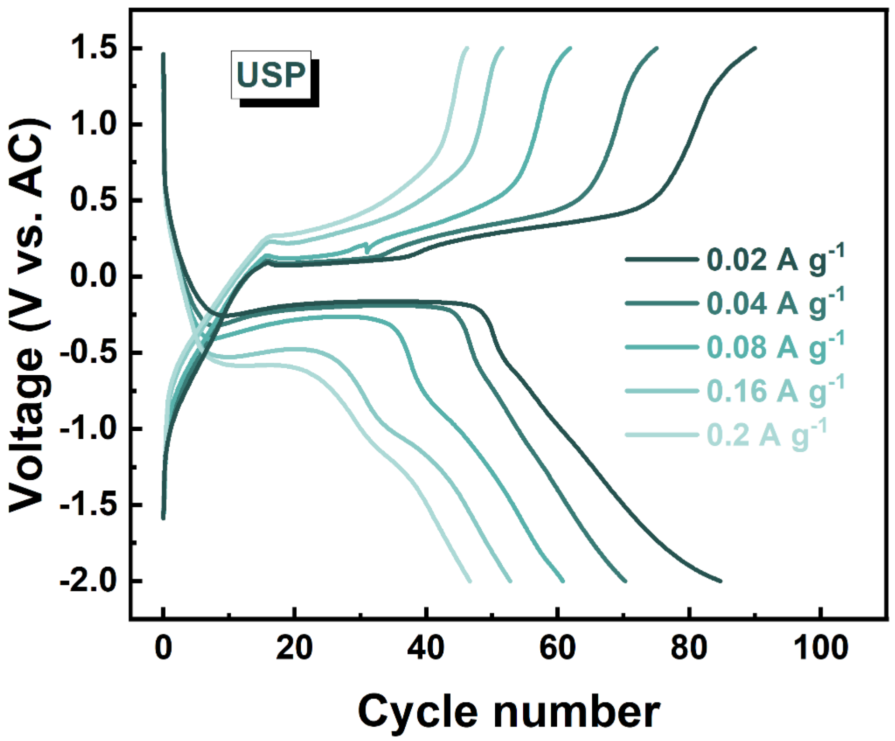

Figure S16. GCD branches at various for solid-solution pyrovanadates USP sample.

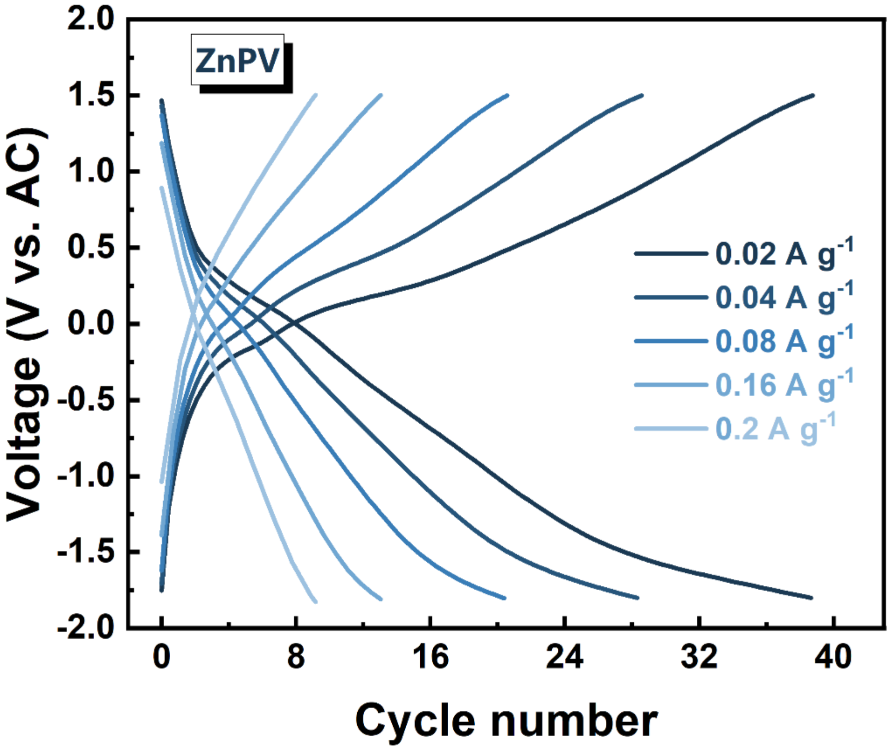

Figure S17. GCD branches at various current densities for single phase pyrovanadates ZnPV sample.

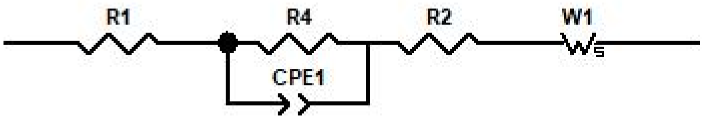

Figure S18. Fitting circuits for EIS analysis.

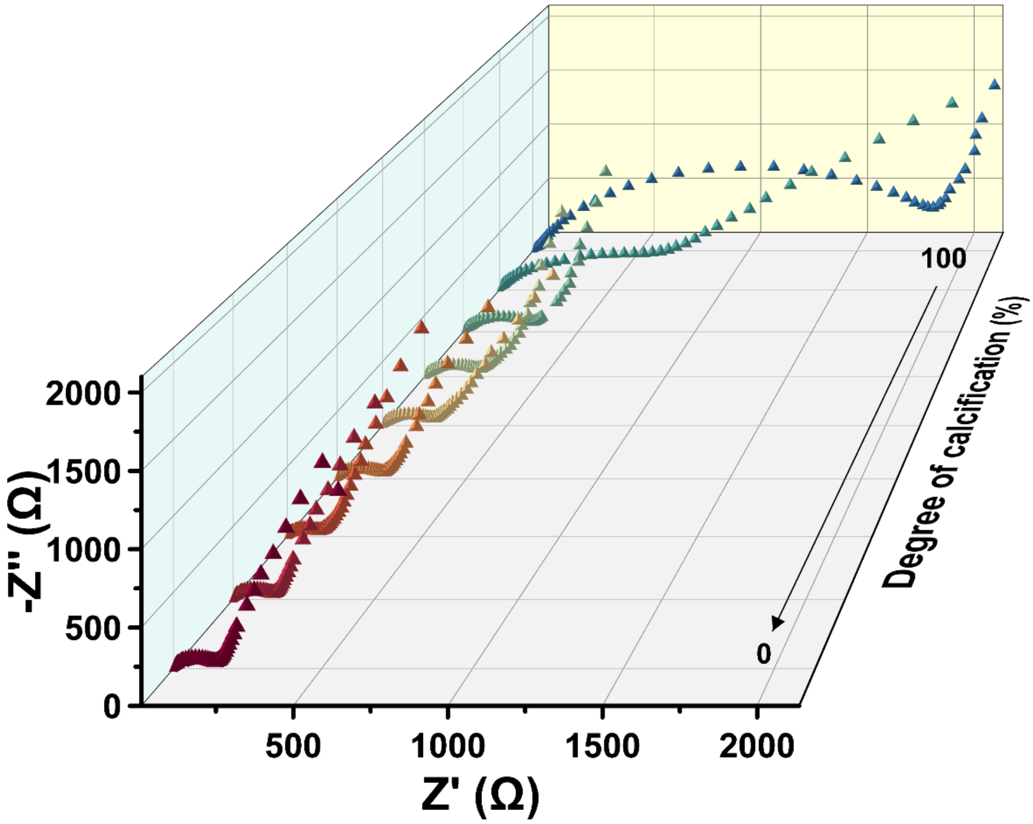

Figure S19. Electrochemical impedance spectroscopy (EIS) during Ca^{2+} ions de-intercalation process.

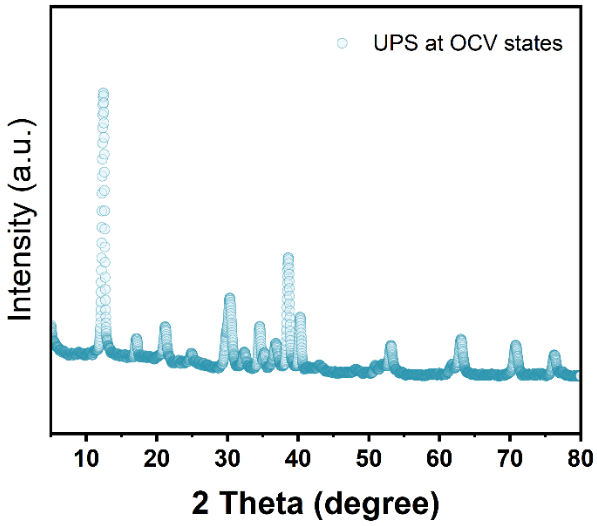

Figure S20. XRD pattern for UPS sample at OCV state.

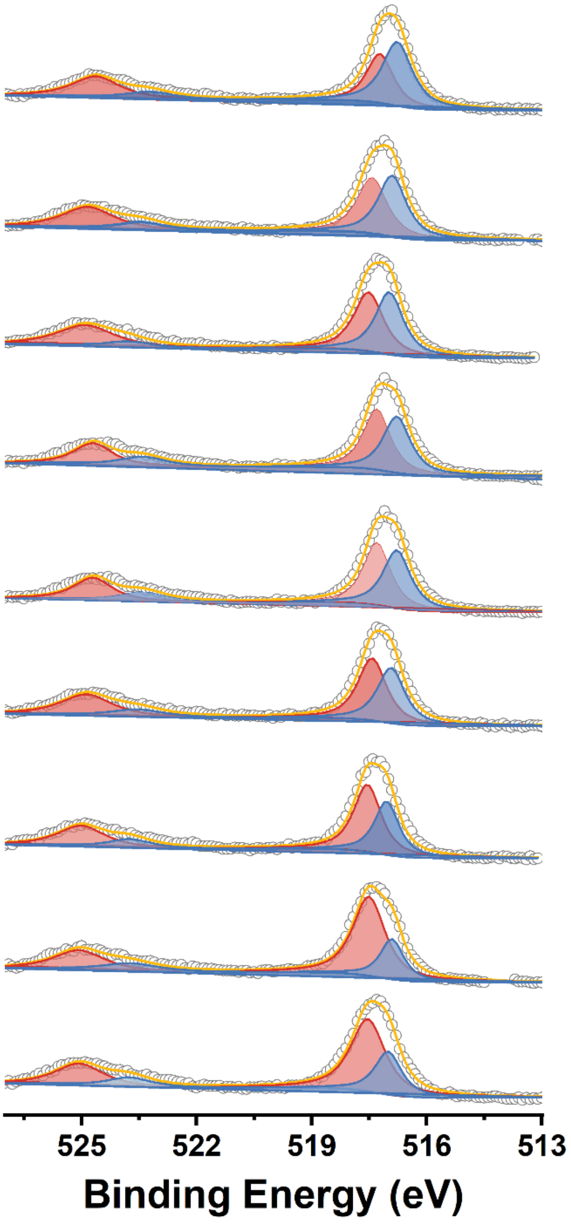

Figure S21. High-resolution V 2p XPS spectra during Ca^{2+} ions de-intercalation process.

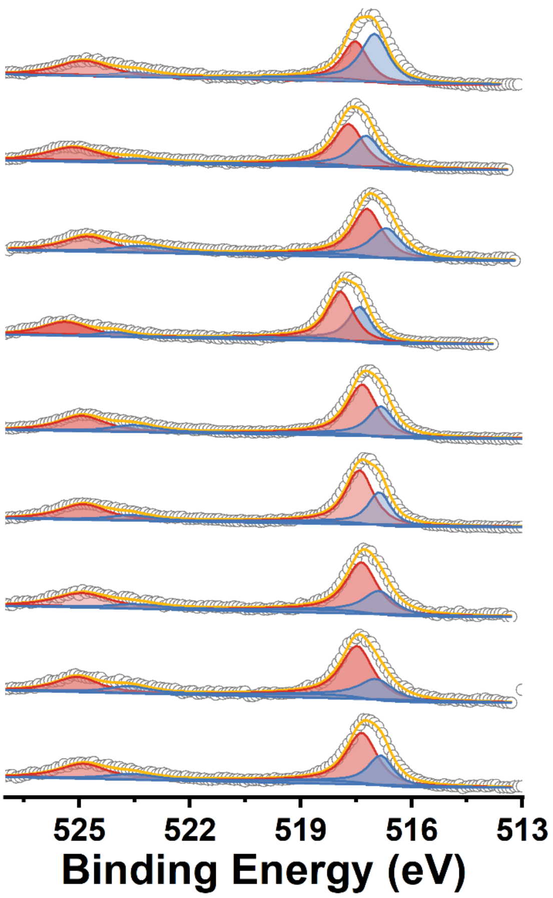

Figure S22. High-resolution V 2p XPS spectra during Ca^{2+} ions intercalation process.

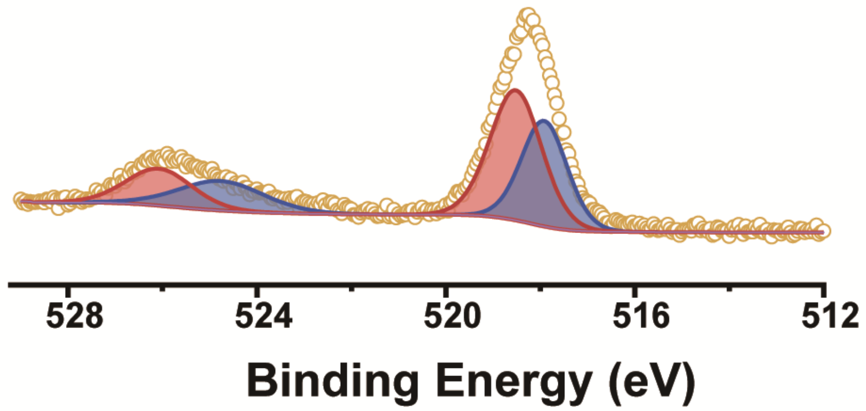

Figure S23. High-resolution V 2p XPS spectra of UPS sample at OCV state.

Table S1 Structural parameters of different samples extracted from the EXAFS fitting. (S_0^2 =0.84)

Sample	Shell	CN	R (Å)	σ^2 (10^{-3}Å^2)	ΔE_0 (eV)	R factor
Zn foil	Zn-Zn	6	2.66±0.01	4.4±3.3	5.2±2.1	0.004
ZnVP	Zn-O	2.9±0.7	1.9±0.01	8.4±8.5	8.4±1.7	0.012
	Zn-Zn	2.6±0.2	3.02±0.01	1.8±1.6	1.7±4.3	
USP	Zn-O	5.3±0.6	1.95±0.01	5.4±2.4	3.6±2.1	0.013
	Zn-Zn	3.1±0.5	3.04±0.01	6.7±7.2	1.2±5.2	
ESP	Zn-O	4.6±0.3	1.93±0.01	4.2±3.1	4.5±3.2	0.013
	Zn-Zn	3.0±0.2	3.01±0.01	2.6±2.2	2.6±5.3	

Data ranges: $3.0 \leq k \leq 12.5$ Å^{-1}, $1.2 \leq R \leq 4$ Å. R: bond distance; σ^2: Debye-Waller factors; R factor: goodness of fit. S_0^2 is the amplitude reduction factor (S_0^2 =0.82 was obtained by foil fitting and applied for the other samples fitting);

Table S2 Structural parameters of different samples extracted from the EXAFS fitting. (S_0^2 =0.71)

Sample	Shell	CN	R (Å)	σ^2 (10^{-3}Å^2)	ΔE_0 (eV)	R factor
V foil	V-V	8	2.60±0.01	1.6±3.2	5.1±2.5	0.006
	V-V	6	3.03±0.01	2.5±2.3	4.1±4.7	
ZnPV	V-O	3.2±0.5	1.70±0.02	4.2±3.1	7.3±5.3	0.017
USP	V-O	3.5±0.4	1.71±0.01	8.4±8.5	2.1±3.2	0.013
ESP	V-O	3.7±0.6	1.72±0.01	5.4±2.4	2.4±5.6	0.009

Data ranges: $3.0 \leq k \leq 12.5$ Å^{-1}, $1 \leq R \leq 2.5$ Å. R: bond distance; σ^2: Debye-Waller factors; R factor: goodness of fit. S_0^2 is the amplitude reduction factor (S_0^2 =0.71 was obtained by foil fitting and applied for the other samples fitting);

Table S3 Structural parameters of different samples extracted from the EXAFS fitting. (S_0^2 =0.84)

Sample	Shell	CN	R (Å)	σ^2 (10^{-3}Å^2)	ΔE_0 (eV)	R factor
Cu foil	Cu-Cu	12	2.54±0.01	8.7±0.7	5.1±0.8	0.004
USP	Cu-O	5.5±0.5	1.91±0.01	6.4±2.6	4.9±3.0	0.013
	Cu-Cu	3.2±0.2	3.13±0.01	11.7±2.3	4.7±2.3	
ESP	Cu-O	4.3±1.0	1.92±0.01	2.4±1.8	2.5±3.6	0.004
	Cu-Cu	3.1±0.6	3.11±0.01	3.7±1.3	2.5±4.3	

Data ranges: $3.5 \leq k \leq 12.5$ Å^{-1}, $1.35 \leq R \leq 3.5$ Å. R: bond distance; σ^2: Debye-Waller factors; R factor: goodness of fit. S_0^2 is the amplitude reduction factor (S_0^2 =0.84 was obtained by foil fitting and applied for the other samples fitting);
